# Matching individual attributes with task types in collaborative citizen science

**DOI:** 10.7717/peerj-cs.209

**Published:** 2019-07-29

**Authors:** Shinnosuke Nakayama, Marina Torre, Oded Nov, Maurizio Porfiri

**Affiliations:** 1 Department of Mechanical and Aerospace Engineering, New York University Tandon School of Engineering, Brooklyn, NY, United States of America; 2 Department of Technology Management and Innovation, New York University Tandon School of Engineering, Brooklyn, NY, United States of America; 3 Department of Biomedical Engineering, New York University Tandon School of Engineerig, Brooklyn, NY, United States of America

**Keywords:** Aptitude, Crowdsourcing, Data quantity, Division of labor

## Abstract

In citizen science, participants’ productivity is imperative to project success. We investigate the feasibility of a collaborative approach to citizen science, within which productivity is enhanced by capitalizing on the diversity of individual attributes among participants. Specifically, we explore the possibility of enhancing productivity by integrating multiple individual attributes to inform the choice of which task should be assigned to which individual. To that end, we collect data in an online citizen science project composed of two task types: (i) filtering images of interest from an image repository in a limited time, and (ii) allocating tags on the object in the filtered images over unlimited time. The first task is assigned to those who have more experience in playing action video games, and the second task to those who have higher intrinsic motivation to participate. While each attribute has weak predictive power on the task performance, we demonstrate a greater increase in productivity when assigning participants to the task based on a combination of these attributes. We acknowledge that such an increase is modest compared to the case where participants are randomly assigned to the tasks, which could offset the effort of implementing our attribute-based task assignment scheme. This study constitutes a first step toward understanding and capitalizing on individual differences in attributes toward enhancing productivity in collaborative citizen science.

## Introduction

Productivity is imperative to success in citizen science, yet retaining participants is a challenge (Chu, Leonard & Stevenson, 2012). Low engagement limits the scope and quality of data (Cox et al., 2015), by hindering the ability of researchers to aggregate data generated by multiple participants (Hines et al., 2015; Swanson et al., 2015). However, a great effort is required to increase participation (Segal et al., 2015) and data volume (Sprinks et al., 2017), especially when the projects focus on specific topics that may not appeal to broad audiences (Prestopnik & Crowston, 2012). A new approach is in need to leverage the effort of limited pools of participants (Roy et al., 2015) and maximize their potential productivity.

A key to the effective use of citizen scientists’ effort may lie in an improved understanding of the varying types of the tasks involved in citizen science (Wiggins & Crowston, 2014). For example, some tasks are designed specifically for data creation, where participants function as distributed sensors to collect data, and others focus on data curation, where they serve as distributed processors to analyze data (Haklay, 2013). Given that each task may require different cognitive abilities, one might enhance productivity by integrating different tasks into a single, cohesive project, where participants are given the choice to opt for a task versus another. A notable example of collaborative citizen science through division of labor is found in iNaturalist (https://www.inaturalist.org), a popular citizen science project with more than 80,000 active participants. In iNaturalist, some participants upload field observations of organisms to the website, and others identify them online. However, the potential benefit of integrating multiple tasks in a single project remains elusive.

Another important aspect may be found in the diversity of participants’ individual attributes. Citizen science projects normally welcome participants who are diverse with regard to experience, demographics, knowledge, and motivation. If any quality or characteristic ascribed to each individual can predict performance in a specific task, it might be possible to harness attributes’ variations toward enhanced productivity via informed task assignment. For example, expertise in the topic is correlated with the level of agreement within and among participants in analyzing geomorphological features of craters on Mars (Wardlaw et al., 2018), and age is correlated with productivity in classifying wild animals online (Anton et al., 2018). Another example of such a correlation is found in the experience in playing action video games. Empirical studies demonstrate that people with the experience tend to perform better in cognitive tasks (West et al., 2008; Dye, Green & Bavelier, 2009; Chisholm et al., 2010; Green, Pouget & Bavelier, 2010). It is suggested that playing action video games could lead to faster processing of visual information (Green & Bavelier, 2003; Green & Bavelier, 2007) or better strategies in completing tasks (Clark, Fleck & Mitroff, 2011). Thus, although the underlying mechanisms are still debatable, the evidence hints at the possibility of informing the division of labor in collaborative citizen science based on experience in playing action video games.

Individual differences in performance can also be explained by variation in motivation to participate. People participate in citizen science projects because of several, diverse drivers, including reputation, collective motivation, norm-oriented motivation, and intrinsic motivation (Nov, Arazy & Anderson, 2011). Among them, intrinsic motivation is found to be a strong predictor for the participants’ performance in citizen science, where participants with high intrinsic motivation are found to be more productive and yield high quality data (Eveleigh et al., 2014; Nov, Arazy & Anderson, 2014; Nov, Laut & Porfiri, 2016; Zhao & Zhu, 2014). Recognizing the diversity of individual attributes among citizen scientists and its correlation to performance, it is tenable to enhance productivity through division of labor in collaborative citizen science by matching individual attributes to task types.

However, it is often difficult to identify which are the individual attributes that can predict performance in specific tasks in advance. The starting point might be literature that provides empirical evidence of the relationship between individual attributes and task performance, grounded in person-environment fit theory (Caplan, 1987). Yet, when the findings in this literature are applied to specific tasks of one’s interest, predictive power may become weaker or even disappear due to many factors, including differences in measurement instruments, low variations in predictor variables, and idiosyncrasy of subject populations. These drawbacks could be alleviated by combining multiple individual attributes to predict task performance. Information fusion is known to produce more informative knowledge by reducing uncertainty, and it has been successfully applied to various fields, such as image processing and sensor networks (Khaleghi et al., 2013). It is thus tenable to enhance the match between individuals and tasks by using multiple individual attributes, even when each attribute has a poor predictive power on task performance.

Here, we investigate the feasibility of enhancing productivity in collaborative citizen science by capitalizing on the diversity of individual attributes among participants. Specifically, we hypothesize that matching individual attributes to task types, informed by literature, will increase productivity in collaborative citizen science. We also hypothesize that combining multiple individual attributes will further reinforce the match between individual attributes and task types, thereby leading to a further increase in productivity. The hypothesis is tested in an image-tagging project composed of two tasks with different granularities: quickly filtering images of interest from an image repository in a limited time, and allocating tags on the object in the filtered images over unlimited time. These tasks are designed to increase efficiency, considering that many image-tagging projects involve analyzing images taken by automated cameras (Lintott et al., 2008; Swanson et al., 2015), which could contain a large amount of images that are of no interest to the researchers. We evaluate the system performance in simulations using real data collected for a citizen science project. We use a project in which a highly polluted canal is monitored as the setting of our experiment, whereby participants are tasked with filtering and tagging real data collected from an autonomous robot deployed in the canal to monitor its environmental health (Laut et al., 2014).

## Theoretical framework

Our study is grounded in two theoretical strands. One is organization theory, in which enhanced group performance is attained by allocating individuals to tasks based on competence, while balancing the effort among tasks (Shafritz & Whitbeck, 1978). Task-specific variations in individual competence are explained by a myriad of personal attributes, including personality (Barrick & Mount, 1991), knowledge (Schmidt, Hunter & Outerbridge, 1986), and age (Veenman & Spaans, 2005). Analogous to enhanced productivity through task specialization (Smith, 1776), adaptive task assignment based on competence can increase overall productivity when the task is decomposable into subsets.

The other is motivation theory, in which different types of individual motivations translate into a certain behavior and performance in combination with task-specific competence (Kanfer, 1990). Motivation is a multifaceted construct, which is broadly divided into extrinsic and intrinsic motivations (Ryan & Deci, 2000). Extrinsic motivation refers to goal-oriented behavioral drivers that come from external sources, such as reward, competition, and compliance, whereas intrinsic motivation is regulated by internal processes, such as enjoyment, curiosity, and inherent satisfaction (Ryan & Deci, 2000). These internal processes are explained by the self-determination theory, which posits people’s inherent growth tendencies in human nature (Deci & Ryan, 2000). In the context of citizen science, volunteers participate in projects through various motivations (West & Pateman, 2016), but the latter is known to be a strong predictor for contribution (Eveleigh et al., 2014; Nov, Arazy & Anderson, 2014; Nov, Laut & Porfiri, 2016; Zhao & Zhu, 2014).

## Materials & Methods

### Setting: our citizen science project

This study was designed as part of the Brooklyn Atlantis Project (Laut et al., 2014), in which an aquatic monitoring robot was developed to take images of the canal along with water quality measurements and upload to our server during the navigation in the canal (Laut et al., 2014). In the past, we have used this project to successfully address various emergent questions in citizen science, including the effects of face-to-face interactions between volunteers and researchers (Cappa et al., 2016), individual curiosity (Nov, Laut & Porfiri, 2016), and interactions with peers (Laut et al., 2017; Diner et al., 2018) on participants’ performance. The specific objective of the project in this study is to allocate tags to the objects of researchers’ interest in the images taken in the canal.

**Figure 1:**
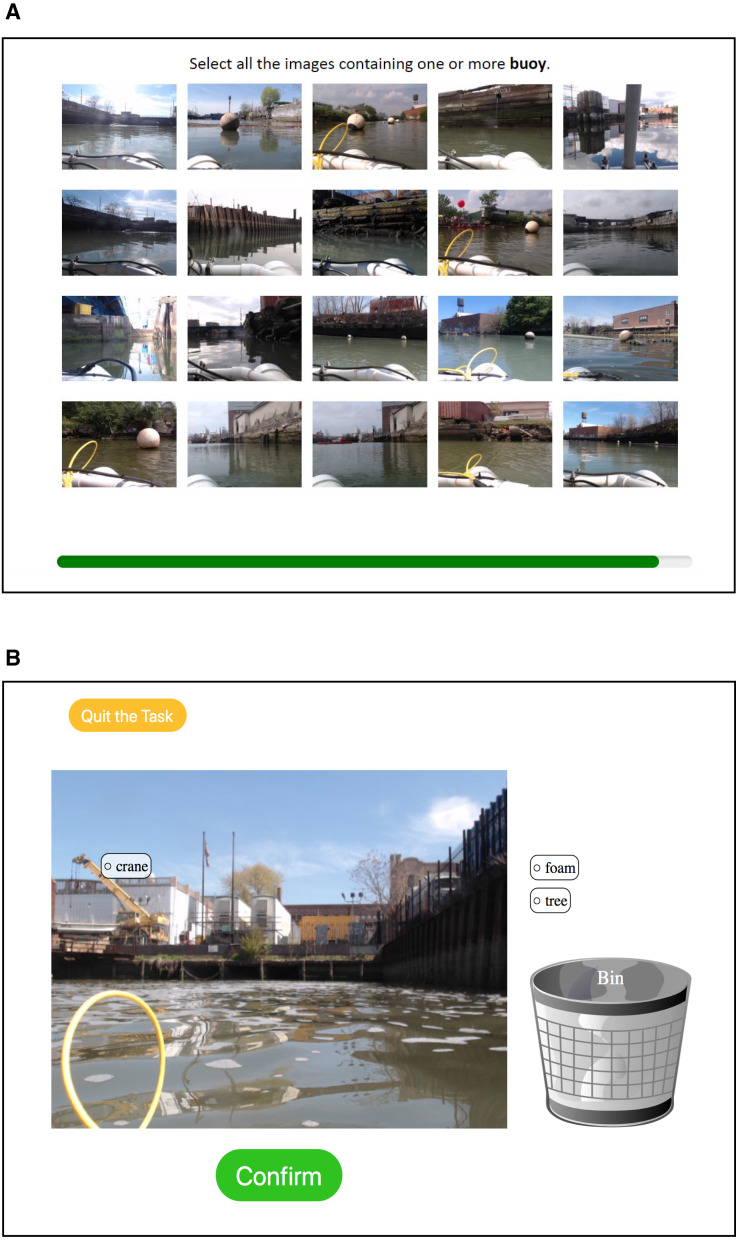
Platform for the citizen science project. (A) Task A, where participants select the images that contain an object of interest within a short time. (B) Task B, where participants allocate the tags to appropriate locations on the image.

The project consists of two tasks: quickly filtering images (Task A) and allocating tags on images (Task B). Task A is designed to filter images that may contain objects of researchers’ interest from an automated image collection performed by the robot (Fig. 1A). A computer screen displays a panel consisting of 20 images, and users select images that contain an object indicated on the top of the panel by clicking them. The selected images are marked by green frames around them, and users can deselect images by clicking them again. Selected images are stored in an image repository with the associated tag names. The same images can reappear for different tags, and therefore, each image in the repository can contain multiple tags.

Task B is designed to allocate image tags on objects in images filtered from the image repository in Task A (Fig. 1B). A computer screen displays an image from the repository generated through Task A, along with associated tags displayed on the side. Users allocate each tag to the object in the image by dragging the tag. When the object indicated by the tag does not exist in the image, users remove the tag by dragging it to the trash bin.

### Experiment

We conducted a controlled experiment using pre-selected images to collect data on individual performance, which were later used to test our hypothesis on matching individuals with tasks. Participants were university student volunteers. Upon agreement to participate by signing a consent form, the experimenter briefly introduced the pollution problem in the Gowanus Canal and our environmental monitoring project.

Next, participants filled in a survey on a computer regarding their motivation to participate in a citizen science activity and their experience in playing action video games. For intrinsic motivation, we asked the following four questions, each of which participants answered on a seven-point Likert scale ranging from ‘Strongly disagree’ to ‘Strongly agree’: (i) *Participation in scientific projects gives me a sense of personal achievement*, (ii) *I really enjoy participating in scientific projects, (iii) Participating in scientific projects is fun*, and (iv) *Participation in scientific projects gives me the chance to do things I am good at* (adapted from Roberts, Hann & Slaughter, 2006). For the experience in playing action video games, we asked participants about the number of hours per day and days per week they spend playing action video games. We did not collect any other personal data, such as age and educational level.

Finally, participants performed both Task A and Task B. In Task A (quickly filtering images of interest), participants were shown nine panels sequentially, with each panel displayed for 5 s. In each panel, participants were asked to select all images that contain the specific object indicated on top of the panel, such as buoy, boat, and tree. Each panel contained 1–8 correct images out of 20 images. In Task B (allocating tags on images), participants were asked to allocate each tag to the appropriate location of the image. Each image was associated with 1–4 tags. Based on a preliminary trial on Task A (*n* = 8), participants incorrectly selected 3% of images as correct. Therefore, in the main experiment, we added 3% of tags incorrectly associated with the image. When they finished allocating all tags on the image, participants clicked a ‘Next’ button on the bottom of the image, and a new image was displayed. Participants continued performing the task until they click a ‘Quit’ button on the screen, or they completed 52 images, the maximum number of images we prepared.

Participants performed Task A and Task B in a random order. Images to both tasks and to all participants were the same. Images were displayed in a same order for all participants in both tasks. The experiment was approved by the University’s Institutional Review Board (IRB-FY2016-184).

### Matching individual attributes with task types

Before examining our hypotheses, we estimated the optimal distribution of participants between the tasks toward maximizing productivity, measured as the total number of tags allocated on the images. To that end, we partitioned the participants into two synthetic groups in a random manner, where one group would perform Task A and the other would perform Task B. We varied the proportions of participants who were assigned to Task A from 0 to 100% with an interval of 10%. We calculated the output in Task A by summing the number of images selected in Task A by the participants who were assigned to the task. In the same way, we calculated the output in Task B by summing the number of tags allocated to images in Task B by the participants assigned to the task. The minimum of the two was used as a measure of the system productivity, considering that the output in Task B is dependent on the output of Task A. By comparing the average system productivity of 10,000 simulations for each proportion, we identified that distributing 40% of participants to Task A and 60% to Task B yielded the highest productivity (2,100 on average).

To assign participants to the tasks based on their individual attributes, we focused on the individual motivation level and video game experience. The individual motivation level was scored as a mean value of the multiple survey responses, and scale reliability was checked by calculating Cronbach’s *α* (Cronbach, 1951). The video game experience was scored as hours playing action video games per week. The motivation and the video game experience were normalized between 0 and 1 by subtracting the minimum value from the observed value and divided by the range, respectively.

We reproduced productivity by dividing the participants into two synthetic groups based on individual attributes. To examine our first hypothesis that using findings in literature could inform better task assignment, we utilized only one attribute to assign tasks to the participants. Specifically, participants were ranked in a decreasing order of the video game experience, and the top 40% were assigned to Task A (quickly filtering images of interest), and the rest was assigned to Task B (allocating tags on images). In a similar way, participants whose motivation fell in the top 60% were assigned to Task B, and the rest was assigned to Task A. In case of ties, we randomly ranked the tied participants.

To examine our second hypothesis that combining individual attributes could improve the process of assigning participants to tasks, the two individual attributes were aggregated into one value as a difference between the two. Specifically, participants were scored as *A* − *wB*, where *A* is the video game experience, *B* is the level of intrinsic motivation, and *w* is a relative weight. The higher score indicates more experience in playing video games, compared to the level of intrinsic motivation. With no a priori knowledge on the relative importance between the two variables on the system productivity, we arbitrarily set *w* = 1. We ranked participants by their scores in a decreasing order and assigned Task A to the participants whose ranks were in the top 40% and Task B to the rest. In case of ties, we randomly ranked tied participants within the ties.

### System evaluation

We evaluated the proposed task assignment scheme by comparing the productivity resulting from attribute-based task assignment against that from random assignment, using the empirical data collected in the experiment. In each simulation, we computed the total numbers of tags allocated on the images in cases where participants were assigned to the tasks randomly and based on individual attributes (motivation only, game experience only, or the combination of both). Then, for each simulation, we recorded the change in the productivity by subtracting the number of processed images through random task allocation from that through attribute-based task allocation. We obtained the probability distribution of the change in output by iterating the simulation for 10,000 times.

In addition, we investigated the relative contribution of the two individual attributes to productivity when they were aggregated into one score to assign participants to the tasks. We evaluated the productivity by assigning participants to the tasks based on individual score *A* − *wB*, where the relative weight of intrinsic motivation on the individual score (*w*) was varied from 0 to 3 with an interval of 0.1, with 10,000 simulations each. The relative contribution of the two individual attributes to productivity was explored by investigating changes in the productivity over *w*.

### Relationships between individual attributes and task performance

Our first hypothesis is built on the empirical evidence of the relationship between the experience in playing action video games and performance in the tasks that require fast visual acuity (West et al., 2008; Dye, Green & Bavelier, 2009; Chisholm et al., 2010; Green, Pouget & Bavelier, 2010), as well as the level of motivation and the quantity of output in citizen science (Eveleigh et al., 2014; Nov, Arazy & Anderson, 2014; Nov, Laut & Porfiri, 2016; Zhao & Zhu, 2014). To ascertain how much these individual attributes would predict task performance in our specific case, we performed a linear regression analysis using data collected from all participants. In one model, we specified video game experience as the explanatory variable and the output in Task A as the response variable. Video game experience was rescaled using an inverse hyperbolic sine transformation to avoid high leverage of large values. In another model, we specified motivation level as the explanatory variable and the output in Task B as the response variable. We tested for the significance by checking improvement of the model fit using an *F* test. Further, to check whether the two attributes were orthogonal to each other, collinearity between the two individual attributes was investigated through Kendall’s rank correlation (Kendall, 1938) between the two individual attributes.

## Results

We collected data from 101 participants. In Task A, participants selected 35 ± 6 (mean ± standard deviation) images among 38 correct images. In Task B, participants allocated 60 ± 40 tags to the images and spent 3.9 ± 2.7 minutes. Eleven participants completed all of the 52 images we prepared in advance. Hours of playing action video games per week ranged from 0 to 28 h (mean 1.2, median 0). The level of intrinsic motivation, estimated as a mean of the responses, ranged from 2.5 to 6 (mean 4.7, median 4.8). Responses from the four questions were highly consistent within participants (Cronbach’s *α* = 0.77).

**Figure 2:**
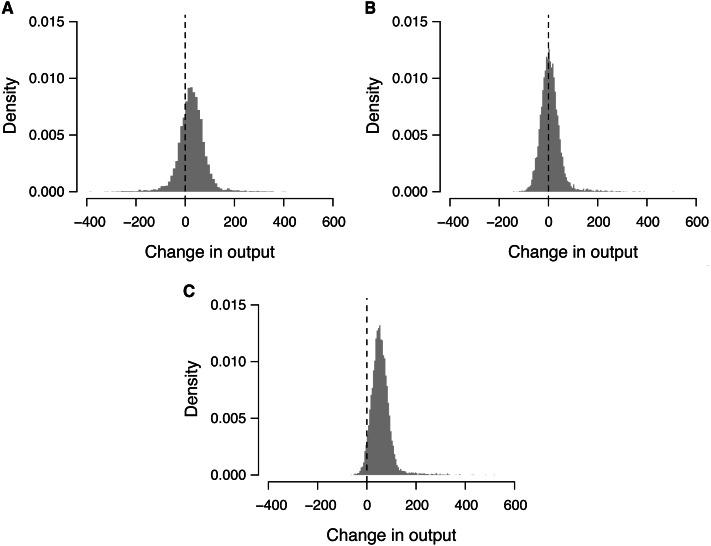
Probability distribution of the change in output through attribute-based task allocation. (A) When participants were allocated to the tasks based only on video game experience, (B) only on motivation, and (C) on both attributes. Change in output was obtained by comparing the number of processed images through attribute-based task allocation against that through random task allocations for 10,000 times. Dashed vertical line represents zero (no change).

When participants were randomly assigned to the tasks, we obtained a productivity of 2, 100 ± 40 (mean ± standard deviation from 10,000 simulations). By contrast, when participants were assigned to the tasks based only on experience in video game playing, we observed a productivity of 2, 126 ± 40. Compared against the random task assignment, it changed the productivity by 27 on average, with a 95% interval from −88 to 130 (Fig. 2A). Similarly, when participants were assigned to the tasks based only on intrinsic motivation, we observed a productivity of 2, 108 ± 13, resulting in a mean change of 8, with a 95% interval from −60 to 101 (Fig. 2B). Finally, when participants were assigned to the tasks based on both attributes, we registered a productivity of 2, 156 ± 5, resulting in a mean change of 56, with a 95% range from −6 to 141 (Fig. 2C). Among these changes, 95.9% cases showed increases from the random assignment, whereas only 3.8% showed decreases.

**Figure 3:**
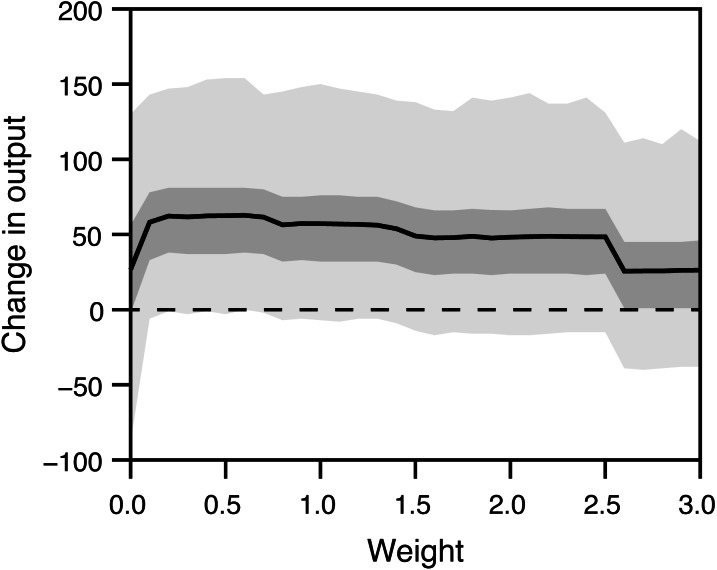
Influence of the relative weight (*w*) on the system output. The participants were ranked by the score *A* − *wB*, where *A* is the experience in playing action video games, and *B* is the level of intrinsic motivation. A solid line represents a mean change in output, and dark and light gray areas indicate 50% and 95% interquantiles of the change in output, respectively, obtained from 10,000 simulations at each value of *w*. A dashed horizontal line is zero (no change).

The weight of the two attributes on individual score influenced the productivity (Fig. 3). The maximum mean change in the productivity (63) was attained at *w* = 0.6.

**Figure 4:**
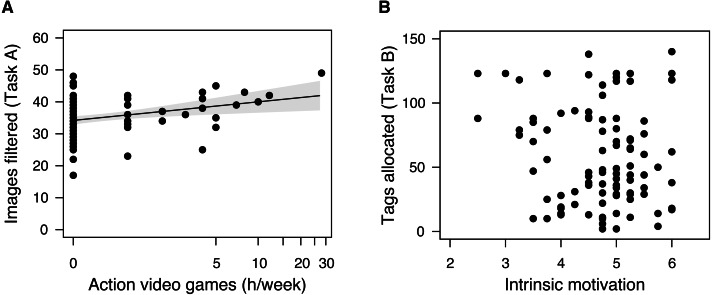
Individual attributes and task output. (A) The experience in playing action video games (h/week) and the number of filtered images in Task A. (B) The level of intrinsic motivation and the number of tags allocated to the images. The experience in playing action video games (h/week) was plotted on a scale of an inverse hyperbolic sine transformation. A line and a shaded area indicates a predicted mean and a 95% confidence band, respectively.

Individual attributes partially explained the task output (Fig. 4). The experience in playing action video game significantly explained the output in Task A (*F*_1,99_ = 9.036, *p* = 0.003). However, the predictive power was low (*r*^2^ = 0.084). By contrast, the level of intrinsic motivation did not explain the output in Task B (*F*_1,99_ = 2.317, *p* = 0.131, *r*^2^ = 0.023). The two attributes were not correlated with each other (*n* = 101, Kendall’s *τ* =  − 0.060, *p* = 0.459).

## Discussion

Our proposed attribute-based task assignment aimed at enhancing citizen science system productivity by capitalizing on multidimensional diversity of human attributes, such that diverse people can contribute collaboratively toward a shared goal. By evaluating the attribute-based task assignment through empirical data, we explored the possibility of enhancing the project’s productivity by integrating multiple weak predictors of task performance in the process of assigning participants to tasks. Our approach of matching individual attributes to task types contributes to designing collaborative citizen science projects that increase system productivity while reducing participants’ effort.

Our proposed task allocation scheme builds on prior empirical evidence that certain individual attributes predict task performance. Specifically, we selected individual attributes that could predict task performance based on empirical evidence: the experience in playing action video games would explain the output of the task that required processing visual information with quick judgment (West et al., 2008; Dye, Green & Bavelier, 2009; Chisholm et al., 2010; Green, Pouget & Bavelier, 2010), and the level of intrinsic motivation would explain the output of the task that required engagement for a prolonged time (Eveleigh et al., 2014; Nov, Arazy & Anderson, 2014; Nov, Laut & Porfiri, 2016; Zhao & Zhu, 2014). In contrast to the literature, however, we found that these individual attributes had extremely weak predictive powers on the task performance in our setting.

The disagreement might have been caused by the experimental procedure, in which we recruited participants on the spot and asked them to perform the tasks on a computer. This situation might have posed a challenge to motivated participants with time constraints, weakening the relationship between motivation and contribution. Alternatively, some people might not have been interested in a local environmental problem. In addition, many of our subject population indicated no action video game playing, which could have weakened the predictive power on the task performance. Nevertheless, we were able to enhance the system productivity by combining two orthogonal attributes in the assignment of participants to tasks, compared to using only one attribute. In addition, combining the two attributes resulted in a lower variation in the productivity, thereby reducing uncertainty of the system output.

The idea of matching individual attributes with task types could be implemented in various crowdsourcing practice. Online crowdsourcing platforms often offer practitioners numerous criteria for selecting workers based on their attributes and experience, which can be used to match workers with specific tasks toward reducing costs by increasing productivity. For example, matching worker expertise and wage requirements with task is shown to enhance knowledge production in collaborative crowdsourcing (Roy et al., 2015). Although many citizen science projects do not collect personal information, it would also be possible to predict individual performance before participants perform tasks by assessing individual attributes through a simple survey. Alternatively, in projects with many recurrent participants, their past performance could also be useful to predict their future performance and assign them to specific tasks. Considering that task performance may be related to a myriad of individual attributes, the idea of combining multiple attributes to inform the selection of which participant should perform which task could find greater applications beyond the case of two attributes we examined here. We believe that such an approach could be effective toward enhancing system performance through an efficient division of labor.

Several factors contributed to enhancing the system productivity by combining the two orthogonal individual attributes. First, dividing participants into dichotomous tasks could alleviate a weak predictive power of individual attributes on task output. The output of each task was estimated as a sum of the output by the participants assigned to the task, and therefore, uncertainty in the output among individuals was mitigated within each task group. By integrating the weak predictors, we could further take advantage of this effect. Indeed, higher productivity was found when the individual attributes were aggregated by weighting less on the level of intrinsic motivation, which had a weaker predictive power. Second, combining the two attributes could differentiate participants with tie scores. As more than half of the participants reported no experience in playing action video games, there was a great uncertainty in assigning participants to the tasks based solely on the video game experience. With additional information of another attribute, we were able to further differentiate individuals within ties, resulting in enhanced system productivity.

It is important to note that implementing attribute-based task assignment into a project can be a significant effort. Although we demonstrated that productivity in the attribute-based task assignment was, in most cases, greater than the values that could be observed by chance, the magnitude of the increase was only 2.7% on average. This is simply due to the fact that the productivity in the attribute-based task assignment is a subset of a random task assignment. As a result, it is not feasible to attain a productivity beyond the upper limit of the null distribution associated with productivity in the random task assignment. It is presently unclear whether such a limited benefit may offset the effort of implementing the attribute-based task assignment scheme into a citizen science platform.

In this study, we explored an idea for designing collaborative citizen science projects that harness variation in individual attributes, using video game experience and motivation as examples. The individual attributes we focused on in this study may show a weaker predictive power in other citizen science projects. For example, if a certain project entails participants of diverse ages, video game experience may not be a valid predictor of a certain task, considering that a relationship between video game experience and cognitive abilities may be confounded by age (Wang et al., 2016). In such a case, practitioners may need to integrate more individual attributes toward accurate task assignment.

## Conclusion

Several citizen science projects offer multiple tasks among which volunteers are free to choose (for example, iNaturalist, https://www.inaturalist.org). Although autonomy in task choices may enhance performance by increasing intrinsic motivation, task preference may lead to an unbalanced distribution of citizen scientists among tasks, thereby diminishing the overall performance in collaborative citizen science. Our study proposes a new direction toward designing citizen science projects toward enhancing productivity through an efficient division of labor that matches individual attributes with task types using multiple individual attributes.

##  Supplemental Information

10.7717/peerj-cs.209/supp-1 Data S1 Data collected from the citizen science project

## Additional Information and Declarations

### Data Availability

The following information was supplied regarding data availability: The data are available in the Supplemental Materials and at GitHub: https://github.com/DynSysLab/BrooklynAtlantis/.
